# CASowary: CRISPR-Cas13 guide RNA predictor for transcript depletion

**DOI:** 10.1186/s12864-022-08366-2

**Published:** 2022-03-02

**Authors:** Alexander Krohannon, Mansi Srivastava, Simone Rauch, Rajneesh Srivastava, Bryan C. Dickinson, Sarath Chandra Janga

**Affiliations:** 1 Department of BioHealth Informatics, School of Informatics and Computing, Indiana University Purdue University Indianapolis (IUPUI), 535 West Michigan St, Indianapolis, IN 46202 USA; 2 Department of Chemistry, The University of Chicago, Chicago, IL USA; 3 Department of Biochemistry and Molecular Biology, The University of Chicago, Chicago, Illinois 60637 USA; 4 Center for Computational Biology and Bioinformatics, Indiana University School of Medicine, 5021 Health Information and Translation Sciences (HITS), 410 West 10th Street, Indianapolis, IN 46202 USA; 5 Department of Medical and Molecular Genetics, Indiana University School of Medicine, Medical Research and Library Building, 975 West Walnut Street, Indianapolis, IN 46202 USA

**Keywords:** CRISPR/Cas13, mRNA regulation, Gene editing, Functional genomics, Machine learning, Protein expression

## Abstract

**Background:**

Recent discovery of the gene editing system - CRISPR (Clustered Regularly Interspersed Short Palindromic Repeats) associated proteins (Cas), has resulted in its widespread use for improved understanding of a variety of biological systems. Cas13, a lesser studied Cas protein, has been repurposed to allow for efficient and precise editing of RNA molecules. The Cas13 system utilizes base complementarity between a crRNA/sgRNA (crispr RNA or single guide RNA) and a target RNA transcript, to preferentially bind to only the target transcript. Unlike targeting the upstream regulatory regions of protein coding genes on the genome, the transcriptome is significantly more redundant, leading to many transcripts having wide stretches of identical nucleotide sequences. Transcripts also exhibit complex three-dimensional structures and interact with an array of RBPs (RNA Binding Proteins), both of which may impact the effectiveness of transcript depletion of target sequences. However, our understanding of the features and corresponding methods which can predict whether a specific sgRNA will effectively knockdown a transcript is very limited.

**Results:**

Here we present a novel machine learning and computational tool, CASowary, to predict the efficacy of a sgRNA. We used publicly available RNA knockdown data from Cas13 characterization experiments for 555 sgRNAs targeting the transcriptome in HEK293 cells, in conjunction with transcriptome-wide protein occupancy information. Our model utilizes a Decision Tree architecture with a set of 112 sequence and target availability features, to classify sgRNA efficacy into one of four classes, based upon expected level of target transcript knockdown. After accounting for noise in the training data set, the noise-normalized accuracy exceeds 70%. Additionally, highly effective sgRNA predictions have been experimentally validated using an independent RNA targeting Cas system – CIRTS, confirming the robustness and reproducibility of our model’s sgRNA predictions. Utilizing transcriptome wide protein occupancy map generated using POP-seq in HeLa cells against publicly available protein-RNA interaction map in Hek293 cells, we show that CASowary can predict high quality guides for numerous transcripts in a cell line specific manner.

**Conclusions:**

Application of CASowary to whole transcriptomes should enable rapid deployment of CRISPR/Cas13 systems, facilitating the development of therapeutic interventions linked with aberrations in RNA regulatory processes.

**Supplementary Information:**

The online version contains supplementary material available at 10.1186/s12864-022-08366-2.

## Background

Gene editing technologies have played an increasingly important role in numerous life science domains in the recent years, especially in the fields of biology, biotechnology, and medicine [1]. At the center of many of these discoveries is the CRISPR/Cas9 gene editing system [2]. This system has allowed an unprecedented level of accurate and precise editing of the genome. Several limitations have been recognized with the use of CRISPR/Cas9 system: the requirement of a PAM (protospacer adjacent motif) sequence adjacent to the target gene sequence, reliance on dynamic DNA repair procedures [3], and its inability to facilitate tissue specific alterations [4]. However, other Cas proteins are being identified and repurposed as systems for genome and transcriptome editing [5].

One such class of protein, Cas13, has been modified to directly edit RNA transcripts [5]. Much like Cas9, the Cas13 system is a two-component system: the Cas13 enzyme and sgRNA. After binding to the sgRNA, the Cas13 complex probes the cellular RNA molecules for a sequence complementary to the spacer sequence of the bound sgRNA. Once identified, the enzyme binds to the RNA molecule for its catalytic cleavage, rendering it ineffective and facilitating RNA degradation. Some of the most promising aspects of this system are the independence from the PAM motif restriction and the potential for designing guide sequences for enabling tissue specific transcript knockdowns.

While the Cas13 system does offer some distinct advantages over the Cas9 system, it also poses some unique challenges. First and foremost, most of the transcriptome remains unknown, owing to poor understanding of various post transcriptional processes. RNA molecules can also adopt a variety of complex three-dimensional structures through networks of inter/intramolecular interactions. This irregular complex structure acts to the number of stretches available for complementary base pairing. Therefore, a tool to predict the efficacy of a given sgRNA is desirable.

**Fig. 1:**
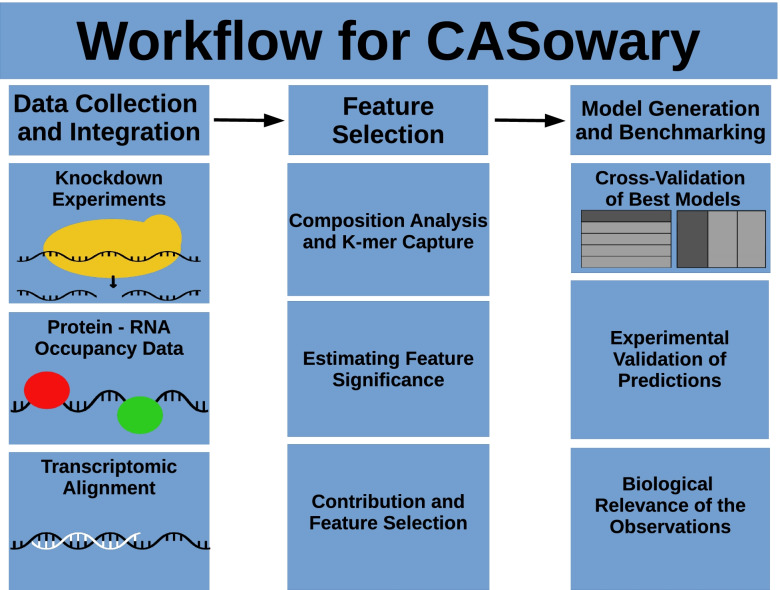
Algorithmic Framework for CRISPR Cas13 Guide RNA Prediction. CRISPR Cas13 knockdown experiments, protein occupancy, and transcriptomic alignment data was gathered for consideration and analysis by the model. Feature lists were created through composition analysis and k-mer capture. The significance and contribution of each feature was estimated to create finalized possible lists of features. The final feature list for the model was generated through comparison of 3-fold and 5-fold cross-validation experiments. Model predictions were validated through direct comparison with performed experiments. The model used to predict sgRNA’s spanning all transcripts associated with 5000 genes. The results were collected and analyzed for any potential biological relevance for predictions

To that end, CASowary was developed as a novel approach for sgRNA efficacy prediction [6, 7]. Although several previous studies have focused on creating software to predict sgRNA for CRISPR Cas9, to our knowledge, there have significantly fewer attempts for doing such for CRISPR Cas13 [8–10]. CASowary was written in python3 and uses a variety of functions from various libraries: vector operations form numpy, statistical analysis from scipy, machine learning utilities from sklearn, and data visualization from seaborn and matplotlib [11–15]. The development and validation of CASowary took place over three distinct phases: Data Collection and Integration, Feature Selection, and Model Generation and Benchmarking (Fig. 1). Three different types of data were utilized by the model for predictions - targeted RNA knockdown experiments [16], transcriptome-wide protein occupancy information [17], and sgRNA spacer sequence alignment data. Feature selection took place through a variety of steps including composition analysis, k-mer capture, and evaluating feature significance and contribution. The model was validated using both 3-fold and 5-fold cross-validation. Additionally, the model’s predictions were verified through an experimental protocol with an orthogonal CRISPR based system [18]. The model was then applied to all transcripts from among 5000 random genes, to determine any biological relevance of the model’s predictions.

## Implementation

### Genome-scale sequence data for CRISPR-Cas13

**Fig. 2:**
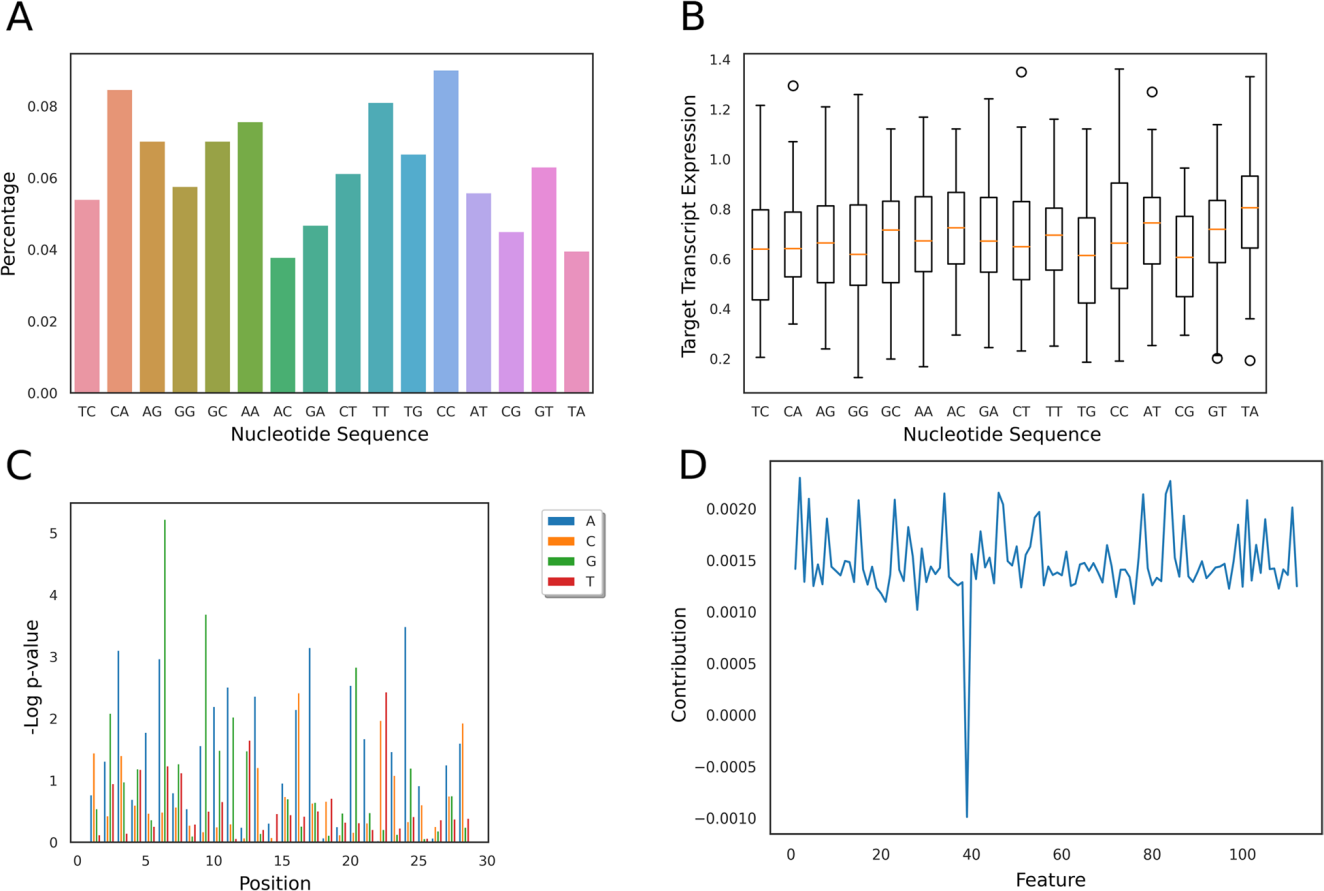
K-mer Analysis to study the guide composition. **A** Bar plot of the population of sgRNAs that contain a specific dinucleotide at position 8. **B** Box plot of target transcript expression values as a function of the nucleotide at position 8. **C** Barplot of negative log of univariate linear regression significance *p*-value for all monomers at all positions across the guide. **D** Bar plot of the feature contribution score for each feature in the Random Forest gini feature list.

Utilizing the Cas13 human transcript knockdown experiments from Abudayyeh et al. [16], we sought to develop a machine learning model that predicts the effectiveness of a given sgRNA at knocking down a target transcript. Firstly, we investigated the sequence composition i.e. mono-, di-, and tri-nucleotide compositions for all 555 guide-RNAs (or sgRNA) at each position along the 28-nt spacer length. We obtained a list of over and under-represented k-mers (chi-squared test) at each location across the spacer sequence of the sgRNA (Fig. 2 A-B). Afterwards, sgRNAs were partitioned into distinct groups based upon their nucleotide composition at a specific location; in order to perform a Kruskal Wallis [19] test. Sets of positions with *p*-values from Kruskal Wallis test less than 0.05 were correlated with nucleotides that were over or under-represented at a particular location (See Supplementary Material).

The significance of each k-mer composition feature was then evaluated using the univariate linear regression module from sklearn. Next, all sequence features with *p*-values greater than 0.05 were removed (Fig. 2C). Wary of being too inclusive with all statistically significant features, two additional subsets of these features were also considered, using a Z-score analysis on the negative log of *p*-values. Using 2 and 3 as the cutoff values, two sets of highly correlated statistical features were generated. In addition to this, a Gini score analysis [20] was performed on each k-mer using the Decision Tree [21] and Random Forest [22] machine learning modules from sklearn. A similar approach was utilized by Fusi et al. [20] for determining the most important features for CRISPR/Cas9 efficiency (See Supplementary Material).

### Protein-RNA occupancy profile

In addition to the sequence composition features, we included transcriptome-wide occupancy as a feature into the model. To do so, we downloaded the transcriptome-wide protein occupancy data (raw reads in the FASTQ format) of HEK293 cells from Schueler et al., SRR1330461 and SRR1330462 [17]. Reads were checked for adapter content and overall quality using Fastqc [23], trimmed using Trim Galore [24, 25], and aligned to the human reference transcriptome,a combination of hg38 cDNA and ncRNA downloaded from Biomart (Additional file 1) [26] using hisat2 [27]. After sequence alignment, peak calling was performed using macs2 (using the -- nomodel option) [28], the resulting .xls file was used as an input for the model (Additional file 2). Each guide sequence was also aligned to the human reference transcriptome, using tophat [29], allowing for 3 mismatches, the maximum number of mismatches tolerated by the CRISPR cas13 system [16]. The indexed position of each guide on the target transcript was compared with the protein occupancy information for any overlap. The amount of overlap was recorded as a percentage of length of the spacer sequence of the guide and incorporated as a feature in the model.

### Additional features

In addition to the k-mer composition and occupancy features, a variety of other features were also included. These include guide spacer percent composition for each nucleic acid, guide location along the length of the transcript, with 0 at the 5` end and 1 at the 3` end, and the observed number of complementary sequences in the reference transcriptome obtained from tophat alignment. A previous study [30] has shown that RNA base composition plays a crucial role in not just the long term stability of the polynucleotide, but also in the activity of the Cas13 system. It is widely believed that the ends of transcripts, both 5` and 3` are highly structured, both to protect the transcript from degradation and to facilitate movement to different cellular compartments. To account for this, relative guide target position was incorporated as a feature into the model by calculating the midpoint of the complementary region of the transcript and normalizing by the length of the target transcript. Finally, in addition to the length and relative position of the spacer sequence with respect to the target, it is possible for a guide to be complementary to multiple regions of the same transcript or portions of different transcripts. This redundancy in targets, could possibly lead to off target effects, and significantly reduce the system’s ability to deplete a target transcript. To capture this in the model, the number of different hits returned from the tophat alignment for each guide was also recorded as a feature.

### Model architecture and feature selection

The occupancy and composition features were then combined with several sets of k-mer features (significant, Z-score > 2, Z-score > 3, decision tree Gini (DT Gini), and random forest Gini) and tested using a variety of machine learning algorithms. Each framework was evaluated based on their ability to accurately classify guides into one of four classes (0–3), based upon the quartile of transcript expression. This was tested by utilizing two different methods of cross-validation: 3-fold and 5-fold. For the 3-fold cross-validation, the experimental replicates were divided into separate folds, with two replicates serving as the testing data, and the other serving as an independent data set. For the 5-fold cross-validation the data from all 3 replicates were randomized, with 80% selected as training data and 20% selected for testing. The average values of the three different 3-fold experiments are presented in Table 1, as well as the average value of 100 different 5-fold experiments.

**Table 1:** Model Architecture Performance by Feature Set

	*P* < 0.05	Z > 2	Z > 3	Gini	Gini DT
3-Fold					
Random Forest	0.717 [55.4]	0.713 [57.55]	0.714 [51.1]	0.716 [54.35]	0.717 [50.55]
KNN	0.715 [2]	0.715 [2]	0.711 [2]	0.717 [3]	0.717 [3]
SVC [linear]	0.71	0.609	0.505	0.6	0.64
SVC [poly]	0.698	0.607	0.517	0.599	0.616
SVC [sigmoid]	0.62	0.551	0.47	0.54	0.553
SVC [rbf]	0.642	0.521	0.487	0.567	0.581
Decision Tree	0.715	0.715	0.711	0.715	0.714
5-Fold					
Random Forest	0.654 [41.3]	0.651 [46.75]	0.641 [45.75]	0.652 [43.95]	0.654 [41.3]
KNN	0.558 [3.39]	0.496 [9.71]	0.515 [8.44]	0.535 [3.67]	0.558 [3.39]
SVC [linear]	0.615	0.553	0.504	0.589	0.634
SVC [poly]	0.593	0.535	0.501	0.562	0.574
SVC [sigmoid]	0.551	0.489	0.458	0.517	0.521
SVC [rbf]	0.563	0.504	0.469	0.537	0.541
Decision Tree	0.634	0.636	0.634	0.64	0.637

Distribution of model accuracy using a variety of different architectures and different feature lists for both 5-fold and 3-fold cross validation methods. For KNN and Random Forest, average values for parameters with the highest accuracy are recorded in brackets

Due to the experimental noise native to the data source methodology, a significant amount of the experimental replicates for a specific guide differed significantly in transcript expression, often by more than 25%. This discordance in the training data lead to the model receiving different labels for the same set of training features, imposing a hard cap to the model’s cross-validation performance. To account for this, the models were evaluated based upon noise-normalized accuracy. The noise-normalized maximum was calculated by counting the number of occurrences where one experimental replicate differed in transcript expression quartile, with another replicate of the same experiment. Put more formally by, computing the size of the set of tuples (i,j) such that x_i_ = x_j_ and y_i_ ≠ y_j_, divided by the size of the set (i,j), and subtracting that value from 1 (where x and y correspond to the model input data and the label, respectively). The total model accuracy was then divided by the noise-normalized maximum to create the noise-normalized accuracy value.

1 $$1-\frac{\left\Vert \left(i,j\right)\vee {x}_i={x}_j\vee {y}_i\ne {y}_j\right\Vert }{\left\Vert \left(i,j\right)\right\Vert }={\mathit{\max}}_{nn}$$

2 $$\frac{acc}{{\mathit{\max}}_{nn}}={acc}_{nn}$$

Once the optimal model architecture and feature set was determined, the importance of each feature was studied. To this end, the model was evaluated using 5-fold cross-validation 100 times, to establish a background. A single feature was removed from the model, and the model was evaluated another 100 times. The difference in model performance between the mean model accuracy and the background was taken to be the result of the removed feature (Fig. 2D).

### POP-seq

Briefly, a total of 20 million cells were subjected to three variants of POP-seq including UV crosslinking, Formaldehyde crosslinking and No-crosslinking approaches (as described in Srivastava et al. [31]). Cells were lysed in trizol and the resulting interphase layer was treated with RNase A/T1, Proteinase K, DNase I followed by depletion of r-RNA.

RNA purity and concentration were assessed at each step using Nanodrop, based on the absorbance ratio 260/280 > 2. RNA integrity was evaluated using Agilent 2100 Bioanalyser system. Atleast 50 ng of r-RNA depleted RNA was used to generate sequencing libraries using the True-seq small RNA library prep kit (Illumina). All libraries were barcoded and sequenced in parallel on a Next-seq platform for 400 million reads to obtain 75 bp single end reads.

## Results

CASowary takes a list of gene names (Additional file 3) as input and exports a list of sgRNA sequences predicted to be at least efficient, with a transcript expression value between 0.5 and 0. The tool first collects a list of Ensembl transcripts that map to the input genes (using Additional file 4), then creates all possible 28 nucleotide guides that span the length of those transcripts and saves them in a FASTA file. The FASTA file is then aligned to the reference transcriptome using tophat, allowing for 3 mismatches, to create a BAM file. The resulting BAM file is converted to a BED file using bedtools [32]. That BED file is then fed into the model where it classifies each guide; and outputs a separate text file for each transcript mapping to an input gene name, containing all highly effective guide sequences ranked upon model confidence in its classification.

Our tool uses a Decision Tree architecture and set of features (Additional file 5) based upon Random Forest Gini analysis to classify a sgRNA into 1 of 4 classes, based upon predicted transcript knockdown efficiency. Each class represents a specific quartile of normalized expression (0: 0–0.25, 1: 0.25–0.5, 2: 0.5–0.75, and 3: 0.75–1). Guides belonging to class 0 and 1 were categorized as highly efficient and efficient, while classes 2 and 3 correspond to inefficient and highly inefficient, respectively. Utilizing 5-fold and 3-fold cross-validation, this model was benchmarked with noise-normalized accuracy of 70.1 and 74.3% respectively (69.2 and 71.5% without accounting for noise in the source data). A small amount of overfitting was observed in the 3-fold cross-validation, due to the identical model inputs, so 70.1% was believed to be the most accurate measure of the model’s performance. The data from the highest performing 5-fold cross validation was saved as the default training data for the published model (Additional file 6).

**Fig. 3:**
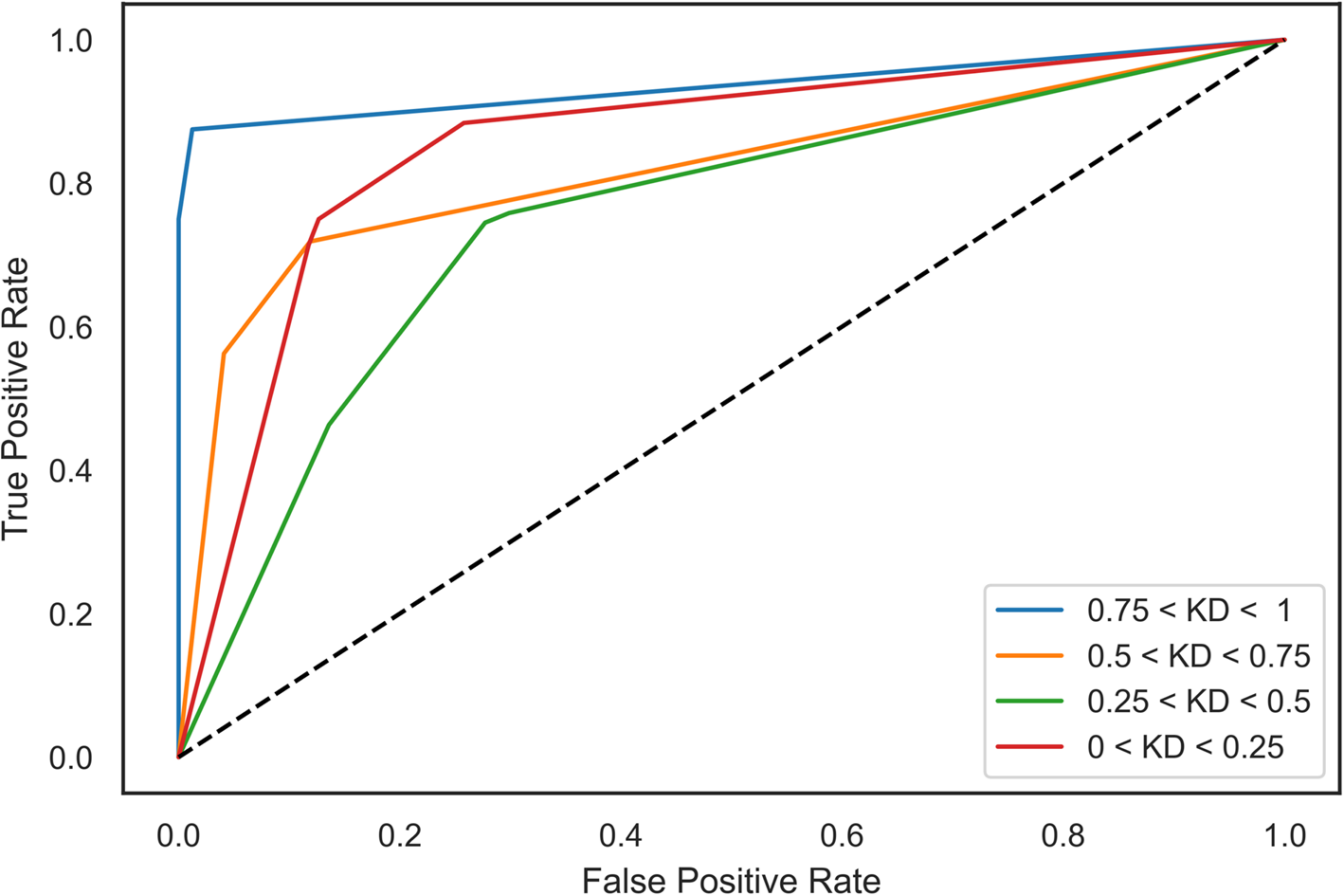
CASowary Model Performance. ROC curve for CASowary Decision Tree model using Random Forest feature list.

Using one class vs all pairwise comparisons, a Receiver Operating Characteristic (ROC) curve for the model was created (Fig. 3). Calculating the Area Under the Curve (AUC) for each class revealed that the model performed best predictions for highly efficient and highly inefficient guides (0: 0.949, 1: 0.869, 2: 0.753, and 3: 0.839). These numbers clearly illustrate an increased sensitivity in model’s predictions for highly efficient and efficient class of guides, maximizing its effectiveness.

**Fig. 4:**
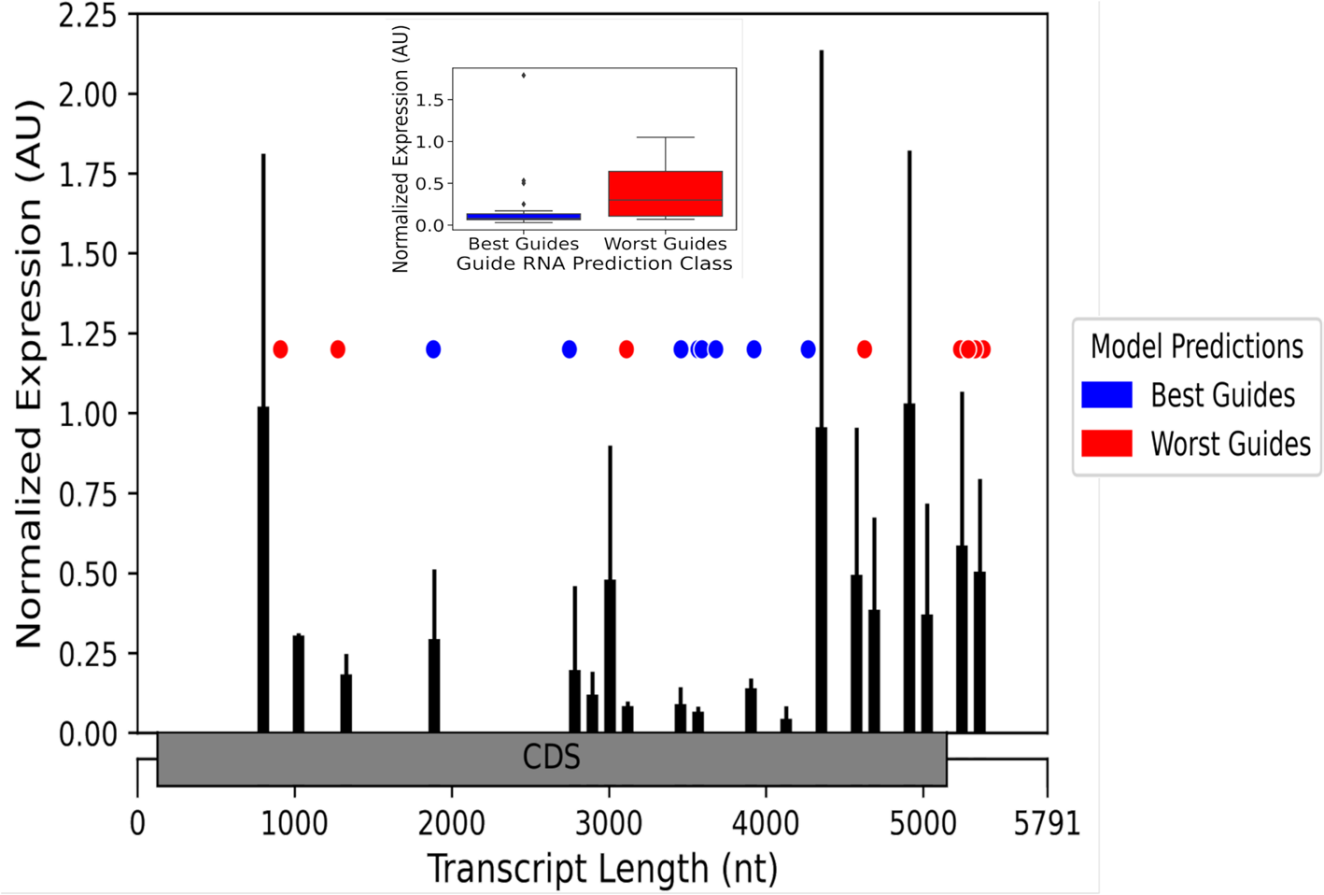
Comparison of CASowary Predictions with CIRTS Results. CIRTS experiments SMARCA4 (add transcript ID) transcript measurements correlated with high efficiency CASowary guide predictions, transcript expression value between 0.25–0 (red) and low efficiency CASowary guide predictions, transcript expression value between 0.75 and 1 (blue)

To confirm the accuracy and robustness of the model, we conducted a series of characterization experiments using an orthogonal RNA-targeting system, CIRTS (CRISPR-Cas-inspired RNA targeting system) [18, 33]. Numerous guides targeting a single SMARCA4 transcript (ENST00000344626.9) were obtained from IDT and the transcript depletion experiment data was generated and analyzed. Comparing our model’s predictions of high (best) and low (worst) efficiency guides and the experimental results of CIRTS showed a very high correlation (Fig. 4).

**Fig. 5:**
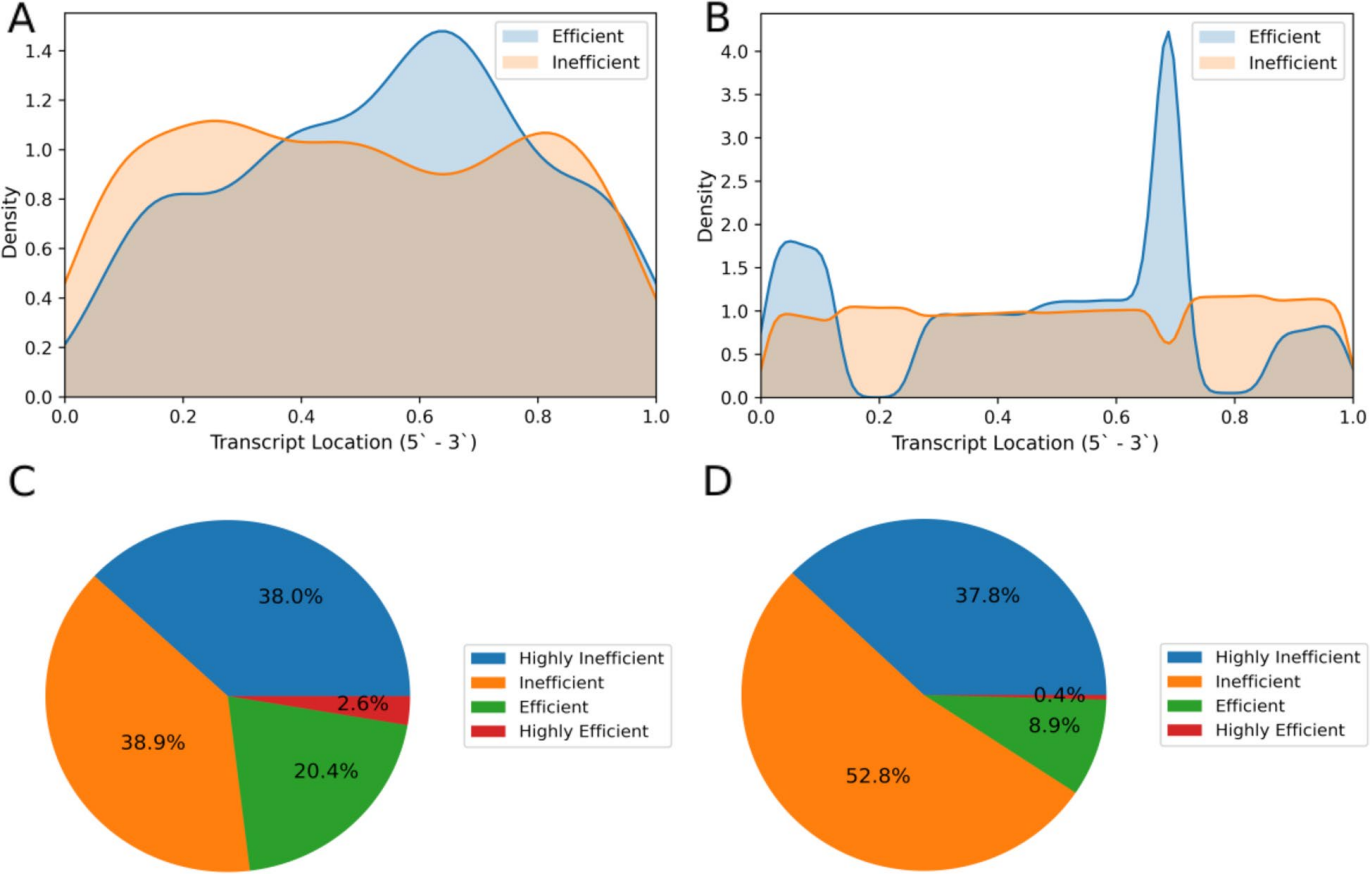
Comparison of Training Data with Gene Predictions: **A** Density plot of Efficient (Highly Efficient and Efficient) and Inefficient (Inefficient and Highly Inefficient) guides from the training data. **B** Density plot of Efficient and Inefficient guides from the 5000 random genes. **C** Pie chart for the breakdown of guide predictions from the training data. **D** Pie chart for the breakdown of the guide predictions from the 5000 random genes

During the development of CASowary, we observed that specific classes of guides exhibited preferential patterns across the length of the target transcript. To confirm this trend, a comprehensive analysis of the predictions for a random assortment of 5000 gene transcripts was performed, resulting in 12.7 million mapped guides. All guides of a specific class were then grouped and plotted against their corresponding location in the transcript, from 5` to 3` direction, by normalizing the length to understand positional preferences for various classes of guides. This analysis revealed that the majority of the guides were predicted to be inefficient, either categorized as Highly Inefficient or Inefficient (90.7%) (Fig. 5 C-D). In addition, our data suggests that efficient guides (Efficient and Highly Efficient) primarily reside in the intermediate regions of the transcript, especially between 30 and 70% the length of the transcript (Fig. 5A-B). Distribution of the guide locations was similar when we plotted the data for the complete training data (Fig. 5A) as well as the computational guide predictions for 5000 genes (Fig. 5B). This observation supports the theory that the ends of active mRNAs are highly structured, that limits the binding efficiency of the CRISPR-Cas13 system.

The secondary location for efficient guides, lying between 0 and 20% of the transcript (near the 5` end) among the computational predictions (Fig. 5B), was unexpected and will require additional investigation. However, the tertiary location for efficient guides, between 0.8 and 1, was of particular interest, due to its lower abundance. Of the 5000 genes included in the analysis, transcripts from 4361 different genes included efficient guides in the upper quintet. That subset included 1417 different genes associated with lncRNA (32%), over 90% of all genes (1570) associated with lncRNA. The average length of these transcripts (1670 nucleotides) was significantly longer than the average length of all transcripts (1537 nucleotides) with *p*-values from Mann-Whitney [34] of 1.34 × 10 ^− 43^. This illustrates that longer transcripts are more likely to have guides in this region, and that this region may be the prime target for lncRNA depletion.

Next, we investigated the ability of CASowary to generate cell type specific guide predictions by employing the tool to predict guide sequences on the HeLa cell line. To this end, we utilized in-house phase separation based protein occupancy data for the HeLa cell line, through a method called Protein-Occupancy Profile Sequencing (POP-seq) to map protein-RNA interactions on a transcriptome wide scale [31]. Protein RNA-interactions are known to vary from cell type to cell type, which would alter the accessibility feature of the current model [35, 36].

The reads from the POP-seq experiment for HeLa cells, corresponding to transcriptomic regions interacting with proteins, were run through the computational pipeline as described in methods (Additional file 7). The resulting file was then substituted for the HEK293 peak file from Schuler et al., in CASowary’s input (see Methods). A list of 100 candidate genes with differential binding profiles between the HEK293 and the HeLa files was generated by running them through DiffHunter [37]. This list of candidate genes was then analyzed using CASowary with the HeLa occupancy profile. Transcript levels were verified by comparing the abundance of reads supporting a specific transcript from RNA-Seq experiments for the respective cell line. This data was obtained for both HEK293 and HeLa cells from the Gene Expression Omnibus (GEO) [38], series accession number GSE146946.

**Fig. 6:**
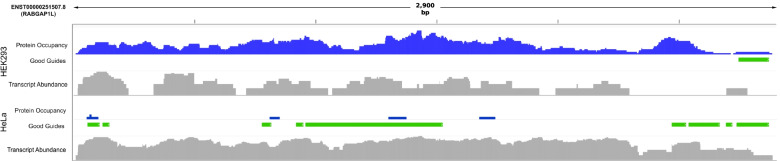
Cell Line Specific Predictions: IGV tracks for ENST00000251507.8, a protein coding transcript for RABGAP1L. The top collection of tracks corresponds to protein occupancy (blue), high quality guide locations (transcript expression value between 0 and 0.5) (green), and transcript abundance for HEK293 cell line (gray). The bottom collection of tracks is the same for the HeLa cell line

The results of CASowary predictions for the two cell lines were visualized using Integrative Genomics Viewer [39] along with relative RNA abundance (SRR11304482 and SRR11304484, for HEK293 and HeLa cells respectively) [40]. We observed that CASowary predicted high quality guides in the transcript regions (ENST00000251507.8 encoding RABGAP1L) that were less occupied by proteins exhibited by the reduced POP-seq signal, thereby indicative of potential guides that can disrupt the RNA transcript (Fig. 6). For instance, in the HEK293 cells, high quality guides were predicted in the end region of the transcript, where there is lower protein binding. While in the HeLa cells, guides were predicted in the start, middle, and end regions of the transcript since there was little to no POP-seq signals detected in these regions. Overall, our results indicate that CASowary can predict high quality guides in a cell type specific manner by employing protein occupancy profiles for the respective cell lines. This observation further illustrates the significance and need for more in-depth protein occupancy protocols to enable guide predictions on gene regulatory regions tailored for specific tissues and cell types.

## Conclusions

Gene and transcript editing technologies, such as CRISPR and its variant systems, will continue to evolve for their application, and so too will the demand for computational and predictive tools to improve the efficacy of these methods. We present CASowary as the first of its kind, tool that provides RNA targeting CRISPR support software. Utilizing the selective set of sequence and RNA accessibility features, our tool can generate a list of potential sgRNAs predicted to be highly efficient, from among thousands of possible guides. Therefore, CASowary’s predictions open the door for new RNA based gene therapies and personalized medicine.

Despite the success of the current iteration of our tool, there still remains room for improvement. In the future, we aim to incorporate additional availability information by considering the structure of the target RNA in vivo. There is also a desire to expand the cell and tissue specific predictions, but that requires substantially more protein occupancy information.

### Availability and requirements

CASowary is written in Python, requiring 3.6.8 or above, with some dependencies on Python 2.7.16. Source code for CASowary is available for free for academic use under GitHub (https://github.com/Janga-Lab/CASowary).

## Supplementary Information

**Additional file 1.** A fasta file containing a complete list of stable Ensembl transcripts for human genome version 38.

**Additional file 2.** A peak file generated using macs2 software on aligned POPPI-seq data for Hek293 cell line using hg38_transcriptome.fasta (Additional file 1) as a reference.

**Additional file 3.** Comma separated file showing the complete list of genes (symbol) used in this study. This forms the basic input for the tool.

**Additional file 4.** List of Ensembl gene IDs, Ensembl transcript IDs, common gene name (symbol), chromosome number, gene start position, gene end position, strand, length of the transcript, and the transcript sequence used in this study.

**Additional file 5 **Tab delimited file containing the k-mer, the position of the k-mer in the guide, and the associated *p*-value for that k-mer.

**Additional file 6.** Tab delimited file containing the training data for the model. The header contains the names for each of the features used.

**Additional file 7.** A peak file generated using macs2 software on the aligned POP-seq data for HeLa cell line using hg38_transcriptome.fasta (Additional File 1) as a reference.

**Additional file 8.**

## Data Availability

All Pop-seq data generated in this study is deposited under GEO accession number GSE166189, and can accessed via https://www.ncbi.nlm.nih.gov/geo/query/acc.cgi?acc=GSE166189.

## Abbreviations

AUC: Area Under the Curve
Cas: CRISPR associated
cDNA: complementary DNA
CIRTS: CRISPR-Cas-Inspired RNA Targeting System
CRISPR: Clustered Regularly Interspersed Short Palindromic Repeats
crRNA: crispr RNA
DNA: DeoxyRibonucleic Acid
GEO: Gene Expression Omnibus
IDT: Integrated DNA Technologies
lncRNA: long non-coding RNA
ncRNA: non-coding RNA
NIH: National Institute of Health
PAM: Protospacer Adjacent Motif
POP-seq: Protein Occupancy Profile sequencing
RBP: RNA Binding Protien
ROC: Receiver Operator Curve
sgRNA: single guide RNA
UCSC: University of California Santa Cruz
